# Antibody-Directed Lentiviral Gene Transduction for Live-Cell Monitoring and Selection of Human iPS and hES Cells

**DOI:** 10.1371/journal.pone.0034778

**Published:** 2012-04-20

**Authors:** Dai-tze Wu, Yasunari Seita, Xia Zhang, Chi-Wei Lu, Monica J. Roth

**Affiliations:** 1 Department of Biochemistry, University of Medicine and Dentistry of New Jersey – Robert Wood Johnson Medical School, Piscataway, New Jersey, United States of America; 2 Deptartment of Ob/Gyn, University of Medicine and Dentistry of New Jersey – Robert Wood Johnson Medical School, Piscataway, New Jersey, United States of America; 3 Department of Pharmacology, University of Medicine and Dentistry of New Jersey – Robert Wood Johnson Medical School, Piscataway, New Jersey, United States of America; Brigham and Women's Hospital, United States of America

## Abstract

The identification of stem cells within a mixed population of cells is a major hurdle for stem cell biology–in particular, in the identification of induced pluripotent stem (iPS) cells during the reprogramming process. Based on the selective expression of stem cell surface markers, a method to specifically infect stem cells through antibody-conjugated lentiviral particles has been developed that can deliver both visual markers for live-cell imaging as well as selectable markers to enrich for iPS cells. Antibodies recognizing SSEA4 and CD24 mediated the selective infection of the iPS cells over the parental human fibroblasts, allowing for rapid expansion of these cells by puromycin selection. Adaptation of the vector allows for the selective marking of human embryonic stem (hES) cells for their removal from a population of differentiated cells. This method has the benefit that it not only identifies stem cells, but that specific genes, including positive and negative selection markers, regulatory genes or miRNA can be delivered to the targeted stem cells. The ability to specifically target gene delivery to human pluripotent stem cells has broad applications in tissue engineering and stem cell therapies.

## Introduction

Human embryonic stem cells (hES) and induced pluripotent stem (iPS) cells are promising resources for gene therapy, drug screening, and regenerative medicine. However, culturing hES and iPS cells is a labor-intensive procedure requiring the enrichment of the pluripotent cells from a heterogeneous population capable of spontaneous differentiation. For iPS cells, a major bottleneck is the low efficiency of reprogramming and the process of identifying and selecting cells reaching the pluripotent state. For hES applications, the ability to drive differentiation toward specific pathways through the introduction of limited factors [1], [2] is of high interest. Subsequent removal of undifferentiated hES cells from a differentiated cell population could avoid the introduction of teratomas into patients. Safe and effective gene delivery is greatly advanced through targeting binding and content release via cell-type specific surface markers. This has been facilitated using lentiviral particles pseudotyped with a modified Sindbis virus envelope, capable of targeting gene delivery using a conjugated antibody [3], [4]. In this study, this system has been adapted for viral entry through cell-surface markers expressed on the hES and iPS cells.

The antibody-directed transduction system utilizes a modified Sindbis virus envelope, termed m 168, pseudotyped onto lentiviral particles [3]. The modifications include the replacement of the laminin binding site with a protein A immunoglobulin G recognition domain (ZZ domain), and serial mutations to suppress heparin-binding sites. The insertion of the ZZ domain allows for targeted viral infection via conjugation with a specific antibody [5]. A variety of antibody molecules have been developed to be effective in targeting specific cell types [6]–[9]. This approach has been successful in targeting cells within a heterogeneous population *in vitro* [9] as well as *in vivo*, where lung metastatic melanoma cells were targeted by m 168-pseudotyped lentiviral particles conjugated with anti-P glycoprotein antibodies through *in vivo* tail vein viral injection [3]. In this study we establish an Ab-mediated transduction system that allows viral entry into hES and iPS cells mediated by antibodies recognizing either the SSEA4 or CD24 surface molecules.

Embryo-derived hES cells offer great hope for their use in therapeutic treatment of various diseases, however ethical concerns regarding these cells remain. Recently, pioneering work indicates that the ectopic expression of transcriptional factors including Oct4, Sox2, Klf4, cMyc, Lin28, and Nanog could reprogram human somatic cells into iPS cells [10]–[15]. During the reprogramming process, fully reprogrammed iPS cell colonies emerge among a large and heterogeneous background population of fibroblasts and incompletely reprogrammed cells. At present, isolation of iPS cells from the heterogeneous population relies on manual selection of colonies via morphological criteria and live-cell staining [15], [16]. Here we describe a robust technique for delivering reporter genes into human iPS cells through the Ab-directed targeted transduction system during reprogramming of somatic fibroblast cells to the pluripotent state. The successfully reprogrammed iPS cells can be specifically infected by the targeting Ab, marked by enhanced green fluorescent protein (eGFP), and enriched under puromycin selection. This provides a relatively easy tool for monitoring and identifying potential iPS cells, as well as hES cells within a mixed heterogeneous population.

## Results

### Optimization of gene transduction using VSV-G pseudotyped lentiviral vectors on the H9 human ES cell line

**Figure 1:**
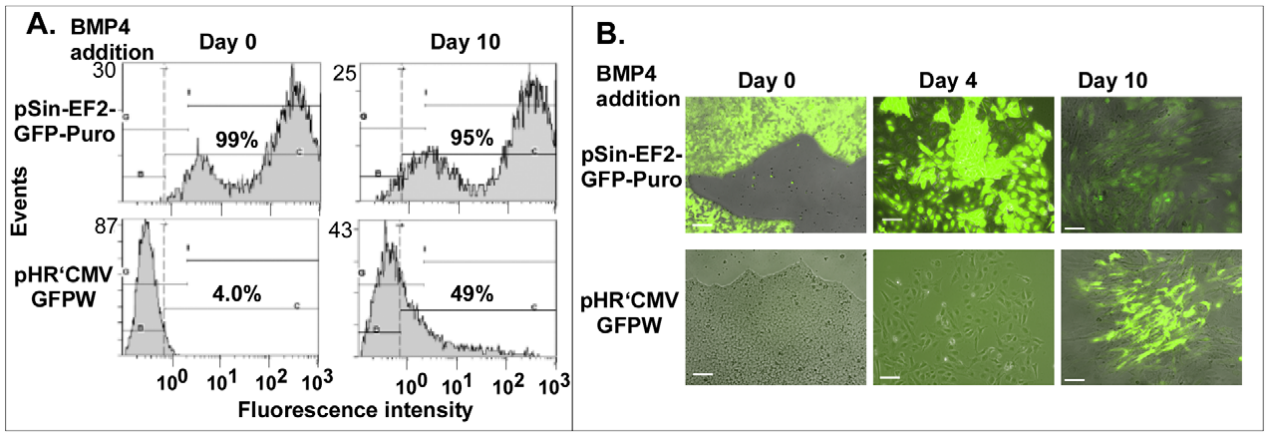
pHR'CMVGFPW (CMV?eGFP? and pSin-EF2-GFP-Puro (EF1α?eGFP?show distinct expressing levels in hES H9 cells and trophoblasts. Trophoblasts were induced from H9 cells by MEF conditioned medium with 20 ng/mL human recombinant BMP4 for 10 days. eGFP. Panel A) Flow cytometry of GFP^+^ cells; Panel B, fluorescence microscopy (100× magnification, scale bar = 100 µm).

Poor viral transgene expression in hES cells is a well-known phenomenon. Conditions were optimized to increase viral infection and expression in the undifferentiated and differentiated hES cells (see Text S1, **Fig. S1 and** **Fig. 1**). Maximal viral transduction was obtained when hES cells were dispersed into single cells with Accutase followed by the addition of the ROCK inhibitor Y-27632 [17] to protect cells from apoptosis and increases colony formation (**Fig. S1**). Variation in the lentiviral vector backbone can also contribute to efficiency of gene transfer and cell expression profiles. Two vectors were compared for expression of eGFP: pHR'CMVGFPW expressed GFP from an internal cytomegalovirus (CMV) immediate-early promoter and pSin-EF2-GFP-Puro expressed GFP from the elongation factor-1 α (EF1α) promoter. Virus bearing both vectors delivered and expressed high levels of GFP into 293T cells (>97% of cells infected; data not shown). In our experimental system, a vector was desired which efficiently expressed GFP in both undifferentiated H9 stem cells as well as BMP4 induced trophoblasts. Fig. 1 compares the eGFP expression from pSin-EF2-GFP-Puro (EF1α promoter) and the pHR'CMVGFPW (CMV promoter) by flow cytometric analysis (panel A) and fluorescence microscopy (panel B) after gene transduction by lentiviral particles pseudotyped with the non-selective VSV-G Env during BMP4 driven trophoblast differentiation of hES H9 cells [18]. The pSin-EF2-GFP-Puro (EF1α promoter) provided maximal GFP expression in both the undifferentiated cells as well as the day 10 differentiated cells with greater than 95% of the cells expressing GFP. In contrast, the pHR'CMVGFPW (CMV promoter) was silenced in the undifferentiated H9 stem cells (4.0% GFP^+^) but active in the differentiated trophoblast cells (49% GFP^+^). Identical results were observed using fluorescence microscopy (Panel B) as with flow cytometry (Panel A). Constructs expressing eGFP from the pSin-EF2-GFP-Puro based vector were used in subsequent studies.

### Specific gene delivery to hES cells via antibody-conjugated m 168 pseudotyped lentiviral vectors

**Figure 2:**
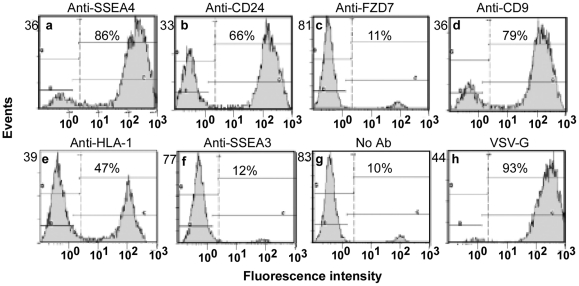
Analysis of antibodies capable of delivering m 168-pseudotyped lentiviral particles into human H9 cells. m 168-pseudotyped lentiviral particles packaging the EF1α-eGFP cassette were incubated with the individual antibodies labeled above each panel and used for infection of human H9 cells. Expression of GFP in H9 cells was analyzed by flow cytometry. Panel a, Anti-SSEA4; Panel b, Anti-CD24; Panel c, Anti-FZD7; Panel d, Anti-CD9; Panel e, Anti-HLA 1; Panel g, Anti-SSEA3; Panel g, No antibody control. In parallel, eGFP delivered by VSV-G-pseudotyped lentivirus was used as a positive control (Panel h).

A key bottleneck in many stem cell applications is the ability to identify, select or counterselect for the stem cells within the mixed population. Specific gene delivery has been achieved using antibody-conjugated systems, in particular lentiviral particles pseudotyped with a modified Sindbis Env (m 168), encoding a protein A immunoglobulin G recognition domain (ZZ domain) [5]. In order to investigate whether the m 168 pseudotyped lentiviral vectors were able to deliver the eGFP gene into the hES cell via a specific monoclonal antibody, we tested a panel of antibodies recognizing hES cell surface marker proteins, including SSEA4, CD24, SSEA3, FZD7, and CD9 (**Fig. 2**) [19]–[21]. Cell surface expression of all the markers were readily detected on the H9 cells by flow cytometry (Fig. **S2**: SSEA3 (93.0%+), SSEA4 (92.3%+), CD24 (99.6%+), FZD7 (77.8%+), CD9 (91.6%+), and HLA-1 (86.4%+)). Transduction efficiency was determined by measuring eGFP gene transfer into hES H9 cells. The results indicate that anti-SSEA4 (**Fig. 2**, panel a, 86% eGFP^+^), anti-CD24 (**Fig. 2**, panel b, 66% eGFP^+^), and anti-CD9 antibodies (**Fig. 2d**, 79% eGFP^+^) conjugated with lentiviral particles pseudotyped with m 168 were able to transduce hES cells. As a control, eGFP delivery in VSV-G-pseudotyped lentivirus was at a level of 93%. Control infection using an IgG k2 isotype antibody resulted in transduction levels equivalent to the no antibody controls, indicating the absence of background from nonspecific transduction of igG antibodies (data not shown). Surprisingly, no transduction was observed using the FZD7 IgG antibody, despite being expressed on the surface of H9 cells (Fig. S2), indicating that not every cell surface protein can serve as an effective receptor for the antibody-mediated transduction [22], [23]. Binding to a receptor is the first step of viral entry leading to a complex series of conformational changes required for membrane fusion and viral content release into the cellular cytoplasm, either at the cell surface or during transport through the cellular endosomal pathways. Variations in the endocytosis and recycling of the cell surface receptors therefore can greatly influence the efficiency of the targeted transduction. Transduction using lentiviral particles conjugated with HLA-1 [3], [9] was 47% eGFP^+^ (**Fig. 2e**).

Antibody binding to the ZZ domain is limited predominantly to IgG molecules. Three of the most frequent used antibodies to identify human embryonic stem cells, anti-SSEA3, TRA-1-60 and TRA-1-81, though are IgM molecules and are predicted not to associate with the ZZ domain [24]. Experimentally, the SSEA3 IgM antibody was not effective in targeting entry, yielding eGFP transduction equivalent to the no antibody control (**Fig. 2** **, panels** **f, g**). A strategy to bridge the SSEA3-IgM complex to the m 168 pseudotyped viral particle via an IgG anti-IgM antibody has failed to rescue targeting (data not shown), indicating a spatial or steric requirement for this targeting strategy.

To further define the specificity, the SSEA4, CD9, CD24, and HLA-1 antibody-mediated transduction using the m 168 pseudotyped lentiviral particles was tested on alternative cell lines. Of critical interest was the ability to recognize human iPS cells, which have been shown to express similar markers as embryonic stem cells. In addition, human foreskin fibroblasts (HFF) and AG1 primary fibroblast (data not shown) were tested as target cells, as fibroblasts are a key source of cells for reprogramming protocols. Using the iPS5 cells line (from Dr. George Q. Daley lab) [14], the transduction efficiency paralleled that of human hES cells for all antibodies tested. Quite significantly, the virus conjugated with the SSEA4 and CD24 antibodies discriminated hES H9 and iPS cells from the differentiated HFF, with an average of 78% and 70% of the hES H9 and iPS cells, respectively, eGFP^+^ after infection in the presence of the CD24 antibody, compared with 1.2% of the cells eGFP^+^ on the HFF (Table 1). The results for the primary fibroblasts AG1 mirrored that of the HFF (data not shown). This differential for infection of hES and iPS cells over fibroblasts was not observed with the CD9 [20] or the general HLA-1 antibody. Thus, gene delivery using the CD24 and SSEA4 antibody-conjugated targeting provides the specificity to infect the hES and iPS cells over fibroblast. All cells positive for infection showed >86% cell surface expression of the marker protein (CD24, SSEA4, HLA-1 and CD9) by flow cytometry. HFF displayed extremely low cell surface expression for SSEA4 (average 3.3%) and CD24 (2.5%).

**Table 1:** Antibody-specific transduction of stem and differentiated cells.

Antibody	Cell line		
	hES H9^a^	iPS5^a^	HFF^a^
No Ab	5.4+/−3.1	6.9+/−2.1	1.6+/−0.47
Anti-SSEA4	73+/−13	80+/−4	10+/−2.1
Anti-CD24	78+/−12	70+/−9	1.2+/−0.15
Anti-CD9	71+/−8	68+/−6.2	73+/−22
Anti-HLA-1	45+/−2	51+/−1.1	67+/−23

a Percentage GFP^+^ cells was determined by flow cytometry after m 168 pseudotyped lentiviral infection. Results are average of 2–3 infections, with average deviations indicated.

### Sensitivity of mAb-mediated selective transduction in a mixed cell population monitored by flow cytometry

**Figure 3:**
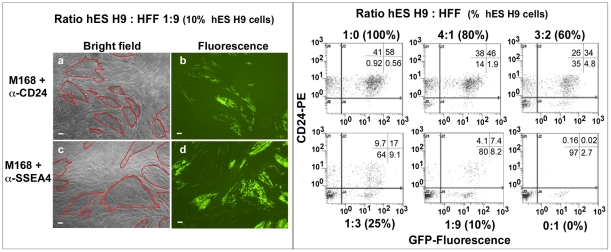
Anti-CD24 and anti-SSEA4 targeted transduction of m 168-pseudotyped virus in a mixed population of hES/HFF cells. Left: Analysis of m 168-pseudotyped viral conjugating with anti-CD24 (left Panels a, b) or anti-SSEA4 (left Panels c, d) into a population of hES H9 cells mixed with HFF at a ratio of 1∶9. Panels a and c, bright field microscopy (40× magnification). Clusters of H9 cells are outlined in red. Panels b and d, fluorescence image of cells expressing GFP (40× magnification). Right panel: eGFP gene transfer by anti-CD24 conjugated viral particles was analyzed on 5 days post-infection by flow cytometry. hES H9 cells and HFF cells were mixed at different ratios (indicated above each panel). Analysis of the hES H9 cells within the population was confirmed by using mouse α-CD24 Ab/α-mouse IgG conjugated with PE. Scale bar = 100 µm.

This differential infection of stem versus differentiated cells was examined within a heterogeneous population, to test whether this method could identify and differentially mark stem cells for specific applications. hES H9 cells and HFF cells were mixed at different ratios and infected by m 168-pseudotyped lentiviral particles conjugated with anti-SSEA4 or anti-CD24 antibodies. **Fig. 3**, left shows the bright field and fluorescence images of the population mixed at 1∶9 ratio of hES H9: HFF cells. For cells infected with the CD24 antibody-conjugated lentiviral particles, GFP expression clustered within cells with the H9 stem cell morphology (panel b). Anti SSEA4 antibodies similarly delivered GFP to H9 cells (panel d), but a background (∼10%, Table 1) of GFP^+^ fibroblast can be observed. The eGFP transduction efficiency was evaluated 5 days post-infection by flow cytometry (**Fig. 3**, right). The level of hES H9 cells within the mixed population was confirmed by flow cytometry using mouse anti-CD24 Ab/α-mouse IgG conjugated with PE. There was a direct correlation of the level of eGFP positive cells transduced through the CD24 Ab-viral conjugated with the percentage of hES H9 cells in the mixed population. In these experiments, maximal antibody-mediated GFP gene delivery corresponded to 58% of the H9 cells (**Fig. 3**, right, 100% H9 cells). These results indicate the lentiviral eGFP gene transduction in the presence of anti-CD24 antibody can specifically label hES within a hES H9 cell/HFF mixed population.

### Targeting and isolation of human iPS cells during reprogramming utilizing anti-CD24 Ab-mediated selective transduction

**Figure 4:**
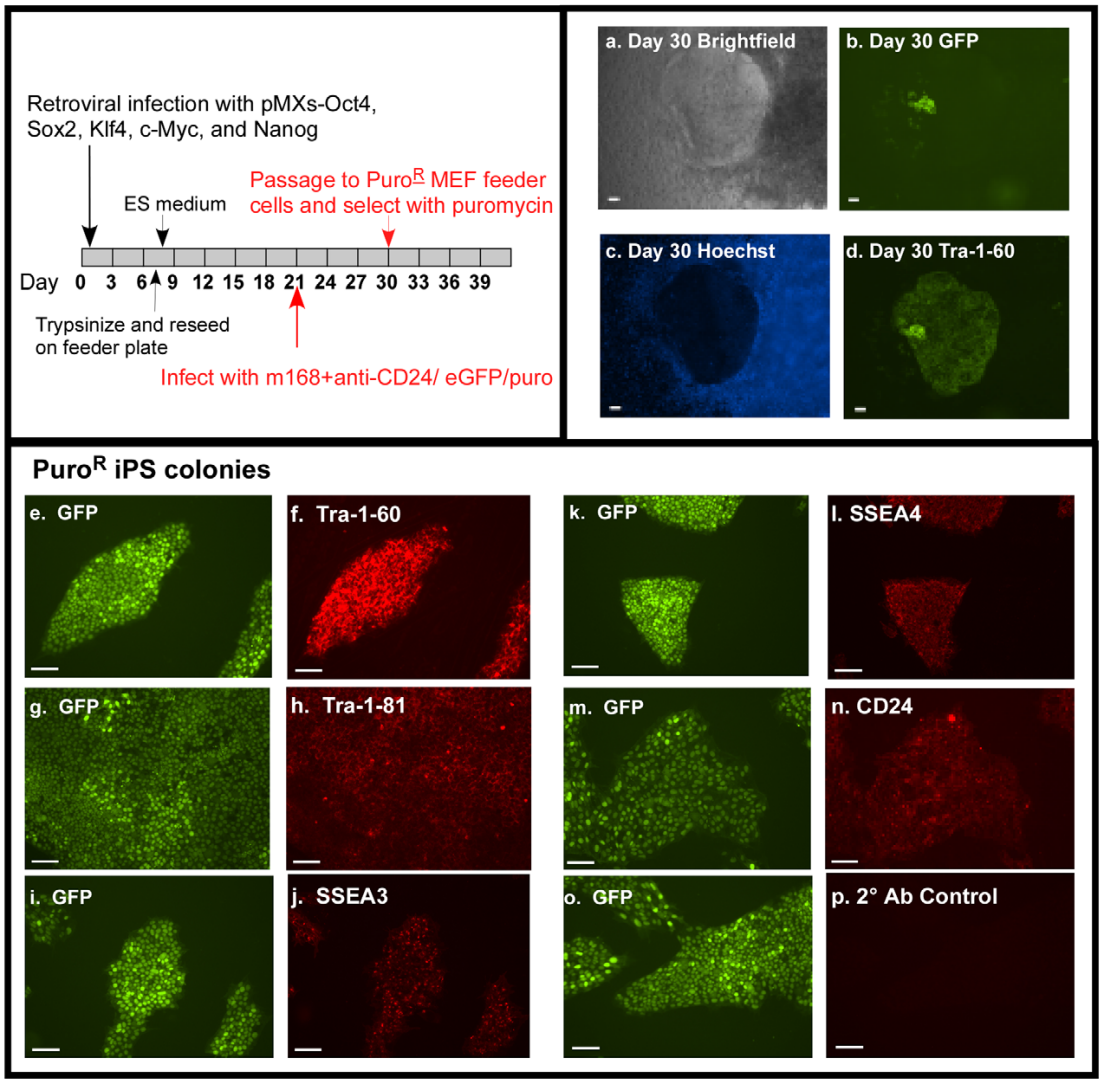
Anti-CD24 directed targeting in reprogramming human iPS cells. Schematic of iPS cell induction, marking and isolation (Top, left). Human primary fibroblasts were infected with pMXs vectors encoding the defined transcriptional factors (Oct4, Sox2, Klf4, c-Myc. Nanog). The cell was then targeted with the anti-CD24 Ab conjugated with m 168-pseudotyped lentivirus 21 days post-induction. Panel a shows the bright field of a putative iPS colony 30 days post-induction. The lentiviral transduced GFP^+^ colonies (Panel b) displayed Hoechst^dim^ (Panel c) and were Tra-1-60 positive (Panel d) in live-staining using DyLight™ 488 conjugated mouse anti-human Tra-1-60. Panels a–d, 40× magnification. Panels e–p, 100× magnification. iPS cells were passaged to Mitomycin treated-^PuroR^MEF feeder cells and selected with puromycin. The selected GFP^+^ iPS colonies (panels e, g, i, k, m, and o) were validated by immunofluorescence staining with Tra-1-60 (Panel f), Tra-1-80 (Panel h), SSEA3 (Panel j), SSEA4 (Panel l) and CD24 (Panel n) using anti-mouse IgG PE conjugated secondary antibodies (Panel l, n) or corresponding Alexa Fluor Series secondary antibodies (Panel f, h, j). Panel p- anti-mouse IgG PE conjugated secondary antibody control (omit primary antibody). Scale bars = 100 µm.

The antibody-mediated gene delivery into cells expressing stem cell markers would be invaluable for the identification of iPS cells during the reprogramming process. The ability of the m 168-pseudotyped lentiviral particles to selectively infect stem cell during reprogramming of human somatic cells to iPS cells was assessed (for timeline, **Fig. 4** top left). Studies were initiated to generate human iPS from African-American human primary fibroblasts by infected with M-MuLV-based retroviral vectors (pMXs) encoding the four defined human transcription factors Klf4, Oct4, Sox2, and c-Myc [12]. In addition, the pMXs-Nanog vector, encoding the monomeric transcription factor Nanog, was included in order to increase the iPS induction efficiency [15]. eGFP-IRES-Puro gene was delivered to iPS cells by anti-CD24 Ab conjugated with m 168-pseudotyped lentivirus 21 days post-induction. By 4 weeks of induction, hES-like colonies (small condensed cells) with low retention of Hoechst dye (Hoechst^dim^) [16], [25] characteristic of undifferentiated human embryonic stem cells were detected expressing both eGFP and TRA-1-60 (**Fig. 4** **a**–**d**). Antibody mediated infection of a preformed colony in the absence of mechanical or enzymatic disruption occurs in a localized patch within the colony, visible by intense GFP staining (Fig. 4 and Fig. S1). Tra-1-60 live staining of the colony using DyLight™ 488 conjugated antibodies indicate a low-level of green labeling of the colony overlapping the GFP+ cells. Dual labeling of eGFP and TRA-1-60 was not observed in cells lacking hES-like morphologies. At 30 days post-induction, colonies were passaged onto puromycin resistance MEF feeder cells and selected by puromycin. After one week of puromycin selection, Puro^R^ iPS colonies were observed which were also enriched for eGFP expression.

Puro^R^ iPS colonies were characterized for their stem cell qualities using multiple assays. Initially, individual GFP^+^ colonies were analyzed for expression of endogenous pluripotent stem cell markers including TRA-1-60, TRA-1-81, SSEA3, SSEA4 and CD24 by immunofluorescence staining and revealed uniform co-expression (**Fig. 4** **e**–**n**). Cells were also positive for alkaline phosphatase (data not shown). Negative control of α-mouse IgG PE conjugated secondary antibody is shown (**Fig. 4** **o, p**); identical results with α-mouse and α-rat IgM Alexa Fluor 594 secondary Abs were obtained (data not shown).

**Figure 5:**
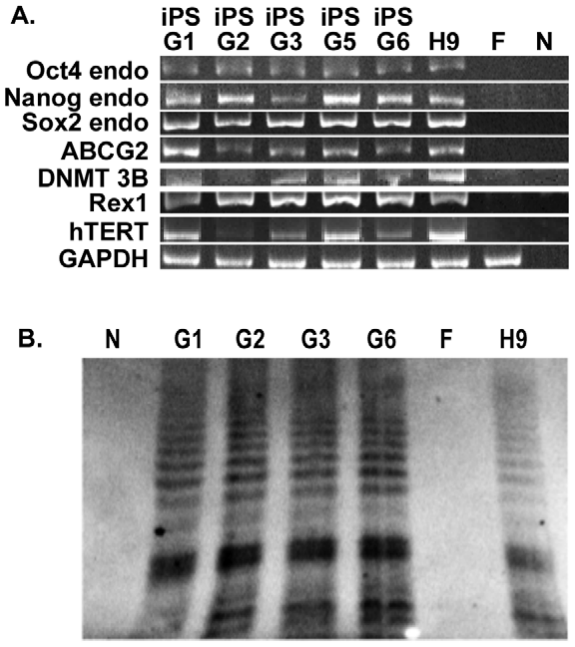
Characterization of endogenous pluripotent makers in selected iPS cell lines. Panel A. Total RNA was isolated using RNeasy Micro Kit from selected iPS cell lines (G1–G3, G5, G6), hES H9 cells (H9), and human primary fibroblasts (F). Total RNA (500 ng) was reverse-transcribed using Superscript III Reverse Transcriptase primed with oligo(dT)_12–18_ and used as template in subsequent PCR with *Taq* DNA Polymerase. PCR analysis examined the expression of endogenous Oct4, Nanog, Sox2, as well as ABCG2, Rex1, DNMT3B and hTERT. GAPDH was used as an internal control. N, no template control (N). PCR products were analyzed on a 10% polyacrylamide TBE Precast Gel. Panel B. TRAP assay for telomerase activity. Selected iPS cells (G1–G3, G6), hES H9 cells (H9), and human primary fibroblasts (F) were analyzed for telomerase activity using the TRAPEZE RT Telomerase Detection Kit as described in M&M. PCR products were separated on 10% polyacrylamide TBE Precast Gel. Individual samples are as indicated.

**Figure 6:**
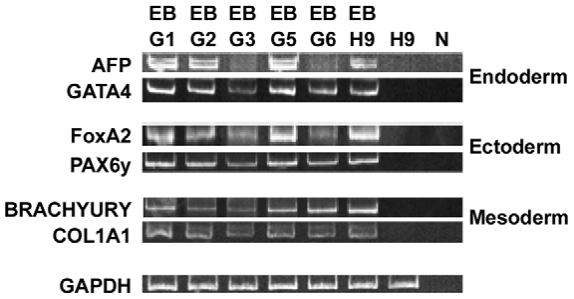
Embryoid Body. EBs from selected iPS cell lines (G1–G3, G5, G6) and hES H9 cells (H9) were harvested 10 days post-differentiation and total RNA (500 ng) was reverse-transcribed and used as template in subsequent PCR reactions. PCR analysis examined the expression of endogenous AFP, GATA (Endoderm), FoxA2, PAX6y (Ectoderm), BRACHYURY, and COL1A1 (Mesoderm). GAPDH was used as an internal control. N, no template control. PCR products were analyzed on a 10% polyacrylamide TBE Precast Gel.

Additional studies analyzed the mRNA levels of endogenous pluripotent makers including Oct4, Nanog, Sox2, ABCG2, DNMT3B, Rex1, and hTERT in five independent iPS cell lines of African American descent selected by the CD24-antibody complexed to m 168 pseudotyped lentiviral particles and puromycin selection (**Fig. 5**). Expression at levels similar to hES H9 embryonic stem cells was detected in the five iPS cells lines. These products were not expressed in the parental primary fibroblasts used to generate the iPS cells (**Fig. 5** **, panel** **A, lane** **F**). A lower level of hTERT was observed in three of the lines (iPS G2, G3 and G6) and telomerase activity was therefore directly measured in these cell lines using the Telomerase Repeat Amplification Protocol (TRAP) assay (Fig. 5, Panel B). iPS G1 with high level of hTERT expression was included as a control for comparison between the PCR and activity assays. High levels of telomerase activity, as judged by the presence of the telomerase repeat products of increasing size was observed in the iPS G1, G2, G3, and G6 cell lines at levels equal or greater than that observed in the hES H9 cells. No telomerase products were detected in the fibroblast control cells. The iPS cell lines were also examined for their ability to differentiate into embyroid bodies and express markers for the three cell lineages (Fig. 6). The expression of markers for the endoderm (AFP and GATA4), ectoderm (FoxA2 and PAX6), and mesoderm (BRACHYURY and COL1A1) using RT-PCR was compared in embryoid bodies (EB) formed from the five iPS cell lines as well as from hES H9 cells. Markers for all three lineages were detected in the EB, which were not present in the undifferentiated H9 cells. We conclude that anti-CD24 antibody mediated selective transduction is effective tool for labeling, selecting and isolating cells with iPS characteristics during reprogramming of fibroblasts.

### Removal of stem cells from differentiated cell populations

**Figure 7:**
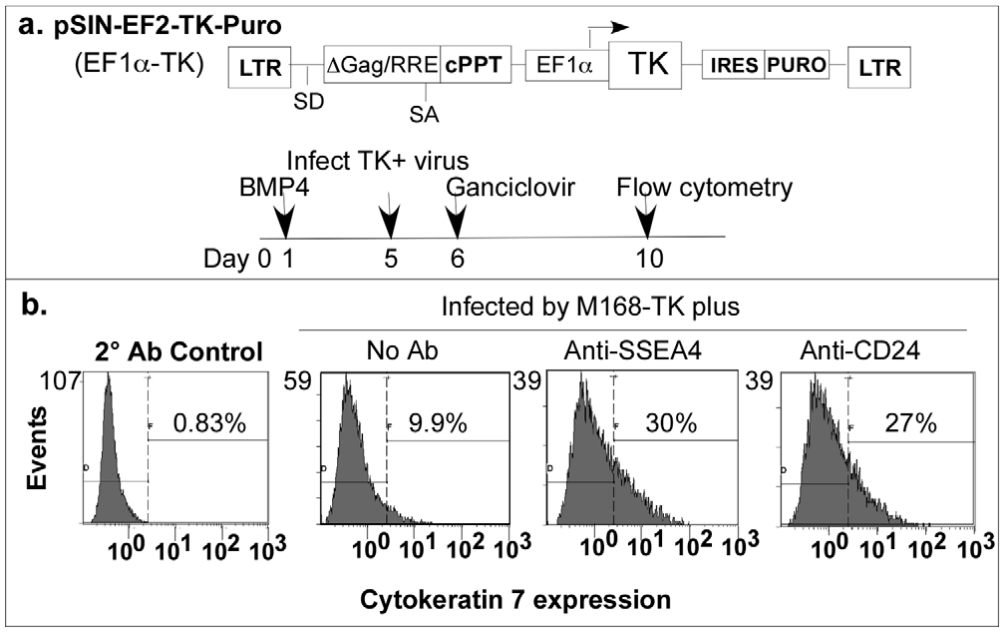
Selective ablation of undifferentiated cells during *in vitro* differentiation. Panel a. Schematic of pSin-EF2-TK-Puro vector and timeline of trophoblast differentiation and selection. hES H9 cells were induced to trophoblasts by culturing in MEF condition medium with 20 ng/mL human recombinant BMP4. m 168 pseudotyped lentiviral particles packaging the pSin-EF2-TK-Puro vector delivered the TK gene into the undifferentiated cells via CD24 or SSEA4 conjugated antibodies five days post BMP4 addition and maintained in 2 µM ganciclovir. Panel b. Trophoblast formation was monitored by flow cytometry for the expression of cytokeratin 7 ten days post BMP4 addition.

The use of the antibody-targeted gene delivery to stem cells offers additional applications for stem cell differentiation protocols. Lentiviral vectors can be used to deliver tissue-specific genes to promote differentiation towards a specific pathway. Alternatively, within any differentiation process, the persistence of undifferentiated stem cells is also a major concern as they can seed teratomas in transplantation recipients. With this goal, the lentiviral vector has been modified to deliver the HSV TK gene, allowing for selective killing of the undifferentiated stem cells in the presence of ganciclovir [26]. **Fig. 7** outlines the vector (pSin-EF2-TK-Puro), with TK driven by the EF1α promoter. hES H9 cells were treated with BMP4 [18], allowing for trophoblast differentiation. Cells were infected with either the SSEA4 or CD24 antibody conjugated m 168 pseudotyped virus bearing the pSin-EF2-TK-Puro lentiviral vector, five days post BMP4 addition and counter-selected with 2 mM ganciclovir. Trophoblast formation was monitored for expression of cytokeratin 7, a marker of both villous and extravillous trophoblasts by flow cytometry ten days post BMP4 addition [27]–[29]. The percent cells expressing cytokeratin 7 increased as a result of ablation of the cells expressing the TK gene through either the CD24 or SSEA4 antibody mediated m 168 lentiviral gene delivery. In the absence of treatment with Ab-targeting virus, 9.9% of the cells differentiated to express cytokeratin 7. In contrast, Ab-targeting through the CD24 or SSEA4 markers with TK+ virus followed by ganciclovir ablation, resulted in 30% and 27% of the cells, respectively, cytokeratin 7 positive. Further analysis of the differentiated cell population indicated the loss of cells expressing high levels of ABCG2, responsible for the (Hoechst^dim^) phenotype characteristic of hES cells [16], [25], proportional to the enrichment observed for cytokeratin 7 positive cells (data not shown). Ganciclovir treatment, in the absence of the TK gene, resulted in a minor decrease of cytokeratin 7+ cells (data not shown), supporting that the enrichment of cytokeratin 7+ cells observed was a result of the loss of TK+ undifferentiated cells.

## Discussion

The undifferentiated state of human ES cells is often characterized by the expression of the cell surface antigens. There are several distinct surface markers expressed on undifferentiated human ES cells, including SSEA4 [30], SSEA3 [31], TRA-1-60, and TRA-1-81 [32]. In addition, CD24, FZD7, and CD9 are also putative human stem cell markers [19], [21]. In this study, we examined the ability of these specific Abs to selectively deliver genes to undifferentiated human ES cell by using the m 168 Sindbis Env antibody bridging system. On the basis of our results, we have demonstrated that lentiviral particles pseudotyped with modified Sindbis virus Envelopes (m 168) containing the immunoglobulin Fc-binding domain were able to target the hES cells and human iPS cells via anti-SSEA4 and anti-CD24 antibodies at a high transduction level with bias for the pluripotent stem cell over fibroblasts. The development of this technology has shown great enrichment of the pluripotent stem cells, enabling rapid and large-scale generation of iPS cells.

Although SSEA4 is a stem cell marker, CD24 is expressed on keratinocytes, mature granulocytes and in many B cells [19], [33] in addition to pluripotent stem cells. For the purpose of identifying iPS cells from skin fibroblasts, this is not a concern because the level of expression on the parental fibroblasts is not significant and thus the induction of CD24 expression on the stem cell is a valid marker for reprogramming. However, if the parental cells used for reprogramming is different, the level of expression of CD24 within this population would need to be verified in order to use this protocol to select for iPS cells.

Tissue specific gene delivery can be achieved through both the viral entry pathway and transcription regulation via specific promoters. The focus of these studies has been the targeted entry of the pseudotyped lentiviral particles, utilizing the EF1α promoter because of its general high level of expression in both the differentiated and undifferentiated cells from hES cultures. Additional layers of selection can be incorporated into this system through the use of stem cell specific promoters. Alternatively, lentiviral vectors in which the reported gene is linked with stage-specific miRNAs have recently been reported to allow for monitoring the reprogramming state of HFF [34]. Combining these transcription/post-transcription systems with the targeted entry would increase the specificity in defining iPS cells in a heterogeneous live cell culture.

The ability to select for the SSEA4 or CD24-targeted cells with puromycin does provide an advantage to enrich and amplify the reprogrammed cells, especially if the efficiency of iPS formation is low. Thus, this provides an alternative to live staining with TRA-1-81 and TRA-1-60 antibodies [15], [16]. It should be noted that the cells transduced within the iPS colony are not characteristically distinct from the other cells, but rather reflects the ability of the virus to infect a pre-formed colony. Morphological selection of putative iPS colonies remains a criteria for passage of cells, and thus distinguishes them from the nonspecific background. In addition, the anti-SSEA4 and anti-CD24 antibody conjugated m 168-pseudotyped lentivirus can discriminate hES cells from mouse embryonic fibroblast (MEF) feeder cells and thus avoid gene delivery into the support cells, if the hES cell lines are cultured on MEF feeder cells. The approach also has the potential to deliver reprogramming factors that may be limiting, allowing for the conversion of reprogramming intermediates to true iPS colonies during the selection process [16].

The use of lentiviral pseudotyped viruses do result in an integrated copy of the transfer vector, and the vectors can be adapted to include the *cre/lox* system to allow for subsequent excision. However, for specific applications, such as the removal of the marked stem cell population from the differentiated population, this is not a concern. For every system, the shortfalls are countered by their benefits. The use of the antibody-conjugated m 168 pseudotyped virus has the potential to both improve the selection and identification of iPS cells, leading to insights into their reprogramming, as well as the studies of hES cells, where delivery of regulatory proteins and selectable markers can improve the homogeneity of the pathways of interest.

## Materials and Methods

### Cell culture

Human H9 ES cell line (WA09) was from the WiCell Research Institute (Wisconsin, USA). iPS5 cells were a gift from Dr. George Q. Daley [14]. hES and iPS5 cell lines were maintained in feeder-free cultures on Matrigel-coated six-well plates (BD Biosciences) with mTeSR™1 (StemCell Technologies Inc). For regular passage, hES cells were treated with 1 mg/mL of dispase for 5 min, collected with a cell scraper and plated. For virus transduction, cells were treated with 1 mg/mL Accutase for 5–10 min until the colonies were dissociated into single cells. The single-cell colonies were then detached and plated with mTeSR^TM^1 containing 5 µM ROCK-inhibitor Y27632 [17] for 24 hr. HEK293T cells purchased from American Type Culture Collection (ATCC; Manassas, VA) and maintained in Dulbecco's modified Eagle medium (DMEM; Gibco) containing 10% fetal bovine serum (FBS; Atlanta Biologicals) and antibiotics & antimycotics (Gibco).

### Plasmids construction

Two vectors were utilized that encoded either the cytomegalovirus (CMV) promoter or the human elongation factor-1α (EF1α) promoter to drive the GFP expression. The CMV-GFP expression vector, pHR'CMVGFPW, was kindly provided by Dr. J. Dougherty (UMDNJ). pSin-EF2-Oct4-Puro (Addgene), encoding the EF1αpromoter was modified to express either GFP (pSIN-EF2-GFP-Puro) or herpes simplex virus thymidine kinase gene (TK) cassettes (pSin-EF2-TK-Puro) through replacement of the Oct4 gene from SpeI to EcoR1. The GFP gene was amplified using pGIP [35] as template with the following primers: EF1α-GFP fwd, 5′-GCA CTA GTG CCA CCA TGG TGA GCA AGG GCG AG -3′; EF1α-GFP rev, 5′-GGC GAA TTC TTA CTT GTA CAG CTC GTC CAT GCC -3′. The TK gene was amplified from the template pAL120-TK (Addgene) using the primers EF1α-TK fwd, 5′- AGC ACT AGT GCC ACC ATG GCT TCG TAC CCCTGC-3′ and EF1α-TK rev, 5′-GGC GAATTC TCA GTT AGC CTC CCC CAT CTC -3′. The SpeI and EcoRI restriction sites are underlined. All of the constructs were verified by DNA sequencing.

The chimeric Sindbis viral envelope vector m 168 was a gift from Dr. I. Chen (UCLA) [3]. The HIV-1 packaging vector pCMV-dR8.2dvpr was purchased from Addgene. The plasmid pHIT-G [36] expresses VSV-G.

### Lentiviral production and transduction

All lentiviral particles were produced by co-transfecting HEK293T cells using Fugene6 (Roche). HEK 293T cells was cultured in DMEM containing 10% ultra-low IgG FBS (Gibco) during the transfection. Briefly, a mixture of 2 µg HIV-based lentiviral expression vector, 2 µg pCMV-dR8.2 dvpr, and 2 µg m 168 vector or pHIT-G, complexed with 18 µL Fugene 6 in 1 mL Dulbecco's modified Eagle medium was added to HEK 293T cells in a 10-cm plate. Supernatants were collected 48 hr post-transfection, filtered through a 0.45 µm pore-size filter and stored at −80°C. Multiplicity of infection was calculated based on the titer of m 168 pseudotyped particles in the presence of the HLA-1 Ab on 293T cells.

H9 cells, iPS cells, HEK 293T, and human foreskin fibroblast cells (2×10^4^ cells) were infected by m 168-pseudotyped lentiviral particles at a MOI of 5 and conjugated with different antibodies (1 µg/mL) for 24 hr. In parallel, cell lines were infected with VSV-G-pseudotyped lentiviral particles as a positive control. The transduction efficiency was detected by the eGFP expression in the target cells using flow cytometry 9 days after infection. Flow cytometry was performed at Rutgers/UMDNJ FACS Core Facility with Beckman Coulter Cytomics FC500 and analyzed using CXP Software.

### Isolation of African-American primary fibroblast cells

The human primary fibroblast cells were prepared as previous reports [37]. The skin punch biopsies were obtained from Cooperative Human Tissue Network, Univ. of Pennsylvania Medical Center. The skin biopsies were washed in DMEM/F12 and minced into approximately 0.5–1-mm size pieces before being seeded onto gelatin-coated 6-well cell culture plates containing mouse embryonic fibroblast (MEF) media consisting of Dulbecco's modified Eagle medium: Nutrient Mixture F-12 (DMEM/F12; Gibco) containing 10% fetal bovine serum (FBS; Atlanta Biologicals) and antibiotics & antimycotics (Gibco).

The culture medium was partially changed every two days. Once the dense outgrowths of fibroblast were expanded to 80–90% confluence (Passage 0; usually day 19–21), the attached biopsy fragments and the fibroblasts were harvested through brief exposure to 0.05% trypsin-EDTA (Invitrogen) and passed through a 70-mm cell strainer to remove large chunks of tissue. These fibroblast cells were cultured until they reached 90% confluence and then frozen in FBS supplemented with 10% dimethyl sulphoxide (DMSO, Sigma-Aldrich) (Passage 1).

### Human iPS cells generation

Human iPS cells were induced by retroviral particles which were produced by co-transfection of the retroviral pMXs vector individually expressing the five transcription factors, including Oct4, Sox2, c-Myc, Nanog, and Klf4 (Addgene) (2 µg) plus the VSV-G envelope vector (2 µg) into TECeB cells [38] by using Fugene (Roche), as recommended by the manufacturer. Viral supernatants were harvested 2 days later, concentrated by Retro-X concentrator (Clontech), resuspended by 5 mL DMEM/F12 with 10% FBS, L-glutamine, 8 µg/mL polybrene (Sigma, H9268), and antibiotic-antimyotic mixture. The virus was used to infect 10^5^ primary fibroblast cells for 24 hours. Seven days post-induction, cells were passaged onto gelatin coated plates which contain mitomycin-treated MEFs cells in iPS reprogramming medium consisting of DMEM/F12 supplemented with 1 mM L-glutamine, 0.1 mM nonessential amino acids, antibiotic-antimyotic, 20% knockout serum replacement (Invitrogen), 0.1 mM β-mercaptoethanol, and 10 ng/ml basic FGF (Stemgent) as previously described [12]. For m 168 Ab-mediated lentivirus transduction, puro^R^-MEF was purchased from StemGent.

### Monoclonal antibodies (mAbs)

Ab-mediated targeting transduction experiments and iPS immunofluorescence staining were performed using the following primary antibodies: Mouse anti-human HLA ABC (Sigma, HLA class I, clone W6/32), anti-SSEA4 (Millipore, MC-813-70), anti-CD9 (Millipore, MM2/57), anti-CD24 (AnaSpec, ML5), TRA-1-60 (Millipore), and TRA-1-81 (Millipore), rat anti-human FZD7 (R&D system, 151143), anti-SSEA3 (Millipore, MC-631). Secondary antibodies used were goat anti-mouse IgG-R-phycoerythrin antibody from Sigma (1∶500), Goat anti-Rat IgG – FITC conjugate, and Alexa Fluor 488 goat anti-rat IgM antibody from Invitrogen. The DyLight™ 488 mouse anti-Human TRA-1-60 antibody (Stemgent) (1 µg/mL) was used for live cell staining of the reprogramming cells by incubating for 30 minutes at 37°C and 5% CO_2_ in the iPS reprogramming medium. Cells were then washed 2 times with iPS reprogramming media and images were obtained on a Nikon Eclipse Ti microscope.

### Trophoblast differentiation and TK negative selection

Before differentiation, 2×10^4^ cell hES H9 cells were treated with Accutase and plated in one Matrigel-coated six-well plate with mTeSR^TM^ containing 5 µM ROCK-inhibitor Y27632. After 24 hr, media was replaced with MEF conditioned medium plus 20 ng/mL BMP4 (Sigma) and changed daily. Five days post BMP4 addition**,** cells were infected with either the SSEA4 or CD24 antibody conjugated m 168 pseudotyped virus bearing the pSin-EF2-TK-Puro (EF1α-TK) lentiviral vector for 24 hours. The infected cells were cultured in MEF conditioned media (CM) medium with BMP4 plus 2 µM ganciclovir (Sigma) from day 7 to day 10. After 4 days selection in ganciclovir, cells were treated with trypsin/EDTA solution, fixed in 2% paraformaldehyde, and permeabilized by suspension in PBS containing 0.1% Triton X-100. 5×10^5^ cells (100 µl of the cell suspension) were mixed with 1 µl of mouse anti-human Cytokeratin 7 antibody (5 mg/ml) (Abcam, RCK105). Secondary antibodies used were goat anti-mouse IgG-R-phycoerythrin antibody from Sigma (1∶500). The samples were analyzed on a FACS Calibur flow cytometer.

### RT-PCR and PCR

Total RNA was harvested using RNeasy Micro Kit (Qiagen) and quantified by spectrophotometer. 500 ng of RNA was used for cDNA synthesis using Superscript III Reverse Transcriptase primed with oligo(dT)_12–18_ (Invitrogen). PCR was performed using *Taq* DNA Polymerase (NEB). Primer sequences were the same as previously described [16].

### TRAP Assay

TRAP Assay was performed by using TRAPEZE® RT Telomerase Detection Kit (S7710 Millipore) with Taq polymerase (NEB), according to the manufacturer's instructions. 500 cells were extracted by CHAPS lysis buffer (S7710 Millipore), extracts were analyzed by PCR with *Taq* DNA Polymerase and separated by 10% polyacrylamide TBE Precast Gel (Bio-Rad).

### EB formation

Human iPS cells were harvested by cell scraper and plated on Ultra low adhesion plate (STEMCELL Technologies) in DMEM/F12 (Gibco) consisting of 15% fetal bovine serum (FBS; Atlanta Biologicals), 15% knockout serum replacement (Invitrogen), 0.1 mM nonessential amino acids and 0.5% penicillin and streptomycin. Media was changed every two day. Ten days post-differentiation, EBs in the supernatant were harvested by centrifugation (BeckmanAllegra-6R, 1000 rpm, 5 min) and RNA was isolated using the RNeasy Micro Kit (Qiagen). Total RNA (500 ng) was reverse-transcribed using Superscript III Reverse Transcriptase primed with oligo(dT)_12–18_ and used as template in subsequent PCR with *Taq* DNA Polymerase. List of primers for amplification of endoderm, ectoderm, and mesoderm markers are included in Table S1.

## Supporting Information

Figure S1 **hES H9 cells disassociated by Accutase improve lentiviral transduction efficiency.** Panels A and C**,** hES H9 cells treated with Dispase followed by the ROCK inhibitor Y-27632. Panels B and D, hES H9 cells treated with Accutase treatment followed by the ROCK inhibitor Y-27632. Panels A and B show the flow cytometry of GFP^+^ cells. Panels C and D show fluorescence microscopy of individual colonies, 40× magnification.
(TIF)

Figure S2 **Expression level of ES H9 cell surface markers.** hES H9 cell surface expression levels were determined by immunofluorescence staining and measured by flow cytometry. The mouse IgG anti-SSEA4, anti-CD24, anti-HLA-1 and anti-CD9 Ab were visualized with anti-mouse IgG PE conjugated secondary antibody. The rat-IgM anti-SSEA3 was visualized with anti-Rat IgM-Alexa488 conjugated secondary antibody. An anti-rat-IgG-FITC conjugated secondary antibody was used for the rat-IgG anti-FZD7 staining.
(TIF)

Table S1 Table of primers for EB differentiation.
(DOCX)

Text S1 Optimization of gene transduction and expression using VSV-G pseudotyped lentiviral vectors on the H9 human ES cell line.
(DOCX)
