# Supplementary figures and images for: Antibody-Directed Lentiviral Gene Transduction for Live-Cell Monitoring and Selection of Human iPS and hES Cells
Source: PLoS One. 2012 Apr 20;7(4):e34778. doi: 10.1371/journal.pone.0034778 (PMC3334894; doi:10.1371/journal.pone.0034778)

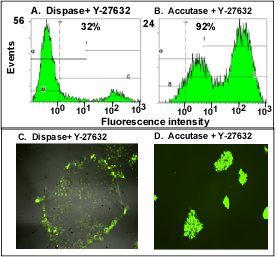

Supplement: Figure S1 — hES H9 cells disassociated by Accutase improve lentiviral transduction efficiency. Panels A and C, hES H9 cells treated with Dispase followed by the ROCK inhibitor Y-27632. Panels B and D, hES H9 cells treated with Accutase treatment followed by the ROCK inhibitor Y-27632. Panels A and B show the flow cytometry of GFP+ cells. Panels C and D show fluorescence microscopy of individual colonies, 40× magnification. (TIF) [file pone.0034778.s001.tif]

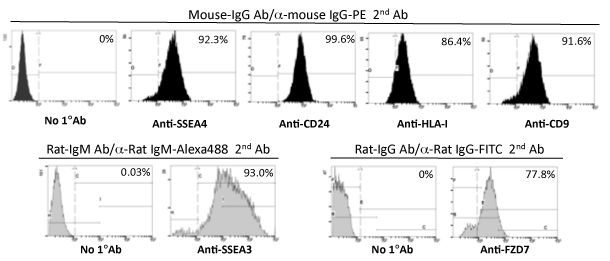

Supplement: Figure S2 — Expression level of ES H9 cell surface markers. hES H9 cell surface expression levels were determined by immunofluorescence staining and measured by flow cytometry. The mouse IgG anti-SSEA4, anti-CD24, anti-HLA-1 and anti-CD9 Ab were visualized with anti-mouse IgG PE conjugated secondary antibody. The rat-IgM anti-SSEA3 was visualized with anti-Rat IgM-Alexa488 conjugated secondary antibody. An anti-rat-IgG-FITC conjugated secondary antibody was used for the rat-IgG anti-FZD7 staining. (TIF) [file pone.0034778.s002.tif]
