# Supplementary material for: Antibody-Directed Lentiviral Gene Transduction for Live-Cell Monitoring and Selection of Human iPS and hES Cells
Source: PLoS One. 2012 Apr 20;7(4):e34778. doi: 10.1371/journal.pone.0034778 (PMC3334894; doi:10.1371/journal.pone.0034778)
Supplement: Table S1 — Table of primers for EB differentiation. (DOCX) [file pone.0034778.s003.docx]

**Table S1. Table of primers for EB differentiation.**

| **Sequence Name** | **Sequence** |
| --- | --- |
| AFPy F | 5’ GAA TGC TGC AAA CTG ACC ACG CTG GAA C 3’ |
| AFPy R | 5’ TGG CAT TCA AGA GGG TTT TCA GTC TGG A 3’ |
| GATA4 F | 5’ CTA GAC CGT GGG TTT TGC AT 3’ |
| GATA4 R | 5’ TGG GTT AAG TGC CCC TGT AG 3’ |
| FOXA2 F | 5’ TGG GAG CGG TGA AGA TGG AAG GGC AC 3’ |
| FOXA2 R | 5’ TCA TGC CAG CGC CCA CGT ACG ACG AC 3’ |
| PAX6y F | 5’ ACC CAT TAT CCA GAT GTG TTT GCC CGA G 3’ |
| PAX6y R | 5’ ATG GTG AAG CTG GGC ATA GGC GGC AG 3’ |
| Brachyury F | 5’ GCC CTC TCC CTC CCC TCC ACG CAC AG 3’ |
| Brachyury R | 5’ CGG CGC CGT TGC TCA CAG ACC ACA GG 3’ |
| COL1A1 F | 5’ AGA CGT TCT CAG TCA GTG CGA TGT 3’ |
| COL1A1 R | 5’ AGG GAA GAG GGA AGA TTA CGC AGT 3’ |
