# Supplementary material for: Antibody-Directed Lentiviral Gene Transduction for Live-Cell Monitoring and Selection of Human iPS and hES Cells
Source: PLoS One. 2012 Apr 20;7(4):e34778. doi: 10.1371/journal.pone.0034778 (PMC3334894; doi:10.1371/journal.pone.0034778)
Supplement: Text S1 — Optimization of gene transduction and expression using VSV-G pseudotyped lentiviral vectors on the H9 human ES cell line. (DOCX) [file pone.0034778.s004.docx]

**Supplemental Text**

**Optimization of gene transduction and expression using VSV-G pseudotyped lentiviral vectors on the H9 human ES cell line.** Conditions were optimized to increase the efficiency of viral transduction into hES cells, which grow in tight and compact colonies. Passage of colonies with Dispase maintains the cells in multi-cell clusters, however viral transduction of eGFP by VSV-G pseudotyped-lentiviral particles into these cells is low (32%) (Fig. S1, panel A). hES cells treated with Accutase results in single cell dispersion, however cell survival is poor due to the induction of apoptosis. Addition of the ROCK inhibitor Y-27632 protects cells from apoptosis and increases colony formation after Accutase treatment^1^**.** hES cells treated with Accutase followed by ROCK inhibitor with subsequent infection of VSV-G pseudotyped lentiviral particles packaging an EF1α-eGFP cassette yielded 92% eGFP^+^ cells (Fig. S1, panel B). Expression of eGFP is high and uniformly expressed throughout the forming colony (Fig. S1, panel D). In contrast, eGFP expression in Dispase treated hES colonies (in the presence of ROCK inhibitor) is heterogeneous, with the highest expression on the periphery of the colony (Fig. S1, panel C). Thus, viral transduction was remarkably enhanced by treatment of the cells with Accutase and ROCK inhibitor one day prior to infection.

1. Watanabe, K. et al. A ROCK inhibitor permits survival of dissociated human embryonic stem cells. *Nature Biotechnology* **25**, 681-686 (2007).
